# Supplementary figures and images for: Pum2 and TDP-43 refine area-specific cytoarchitecture post-mitotically and modulate translation of Sox5, Bcl11b, and Rorb mRNAs in developing mouse neocortex
Source: eLife. 2022 Mar 9;11:e55199. doi: 10.7554/eLife.55199 (PMC8906809; doi:10.7554/eLife.55199)

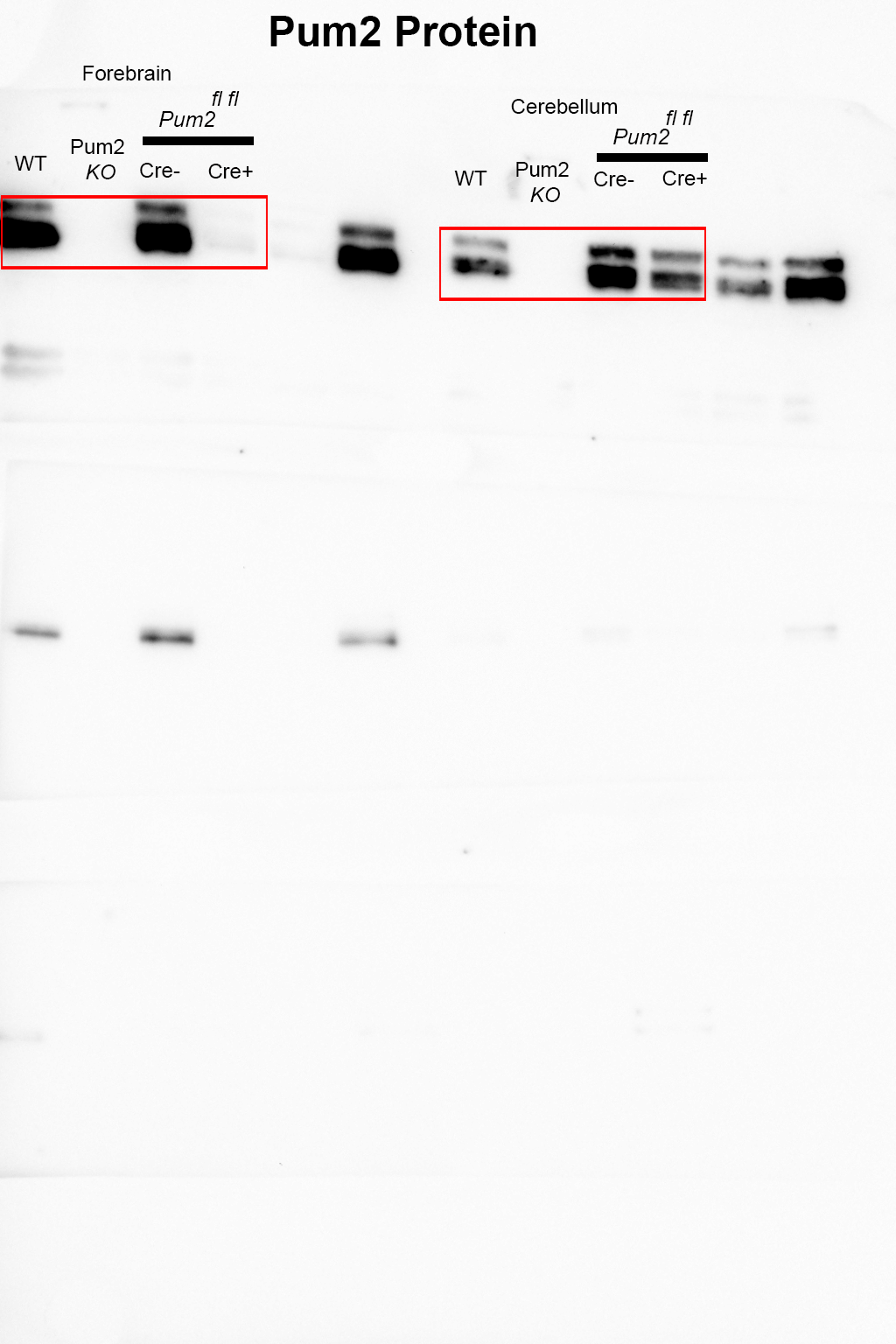

Supplement: Source data 30. — A zipped folder containing original and labeled bands photos for Western blots of Pum2 and tubulin as control (Figure 1—figure supplement 1e), human and mouse TDP-43 and total protein stain as control (Figure 1—figure supplement 8c), and Emx1 and total protein stain as control (reviewers Figure 1b). [file elife-55199-data30.zip › WB original and labeled blots/Fig1-Fig Suppl 1e Pum2 labeled blot.tif]

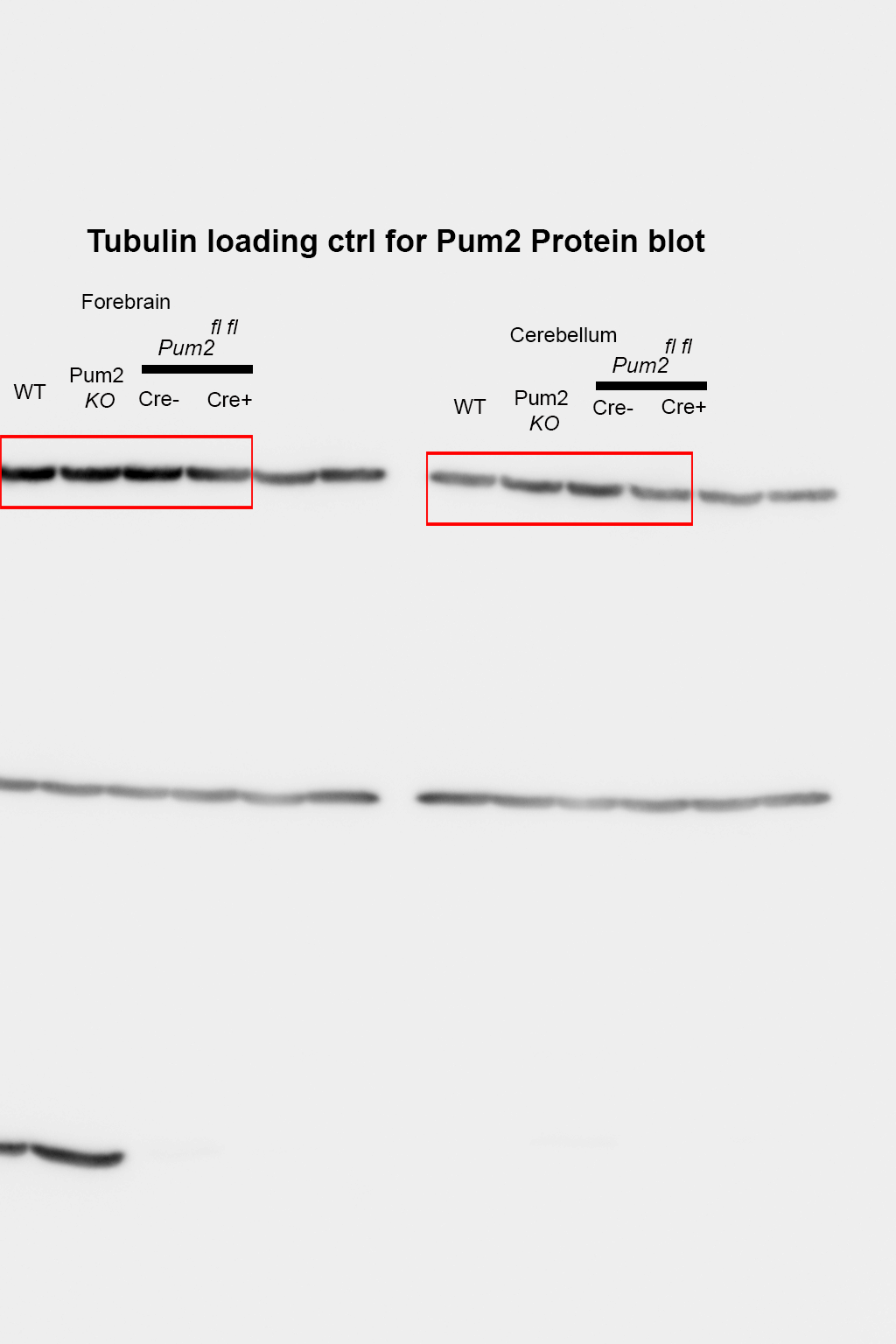

Supplement: Source data 30. — A zipped folder containing original and labeled bands photos for Western blots of Pum2 and tubulin as control (Figure 1—figure supplement 1e), human and mouse TDP-43 and total protein stain as control (Figure 1—figure supplement 8c), and Emx1 and total protein stain as control (reviewers Figure 1b). [file elife-55199-data30.zip › WB original and labeled blots/Fig1-Fig Suppl 1e Tubulin labeled blot.tif]

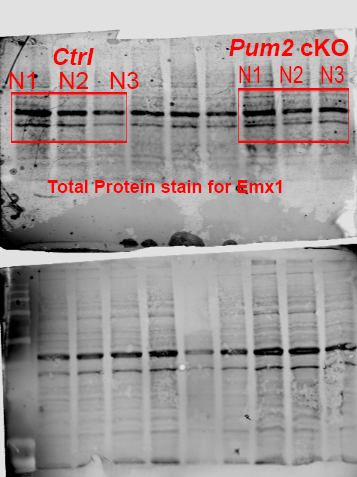

Supplement: Source data 30. — A zipped folder containing original and labeled bands photos for Western blots of Pum2 and tubulin as control (Figure 1—figure supplement 1e), human and mouse TDP-43 and total protein stain as control (Figure 1—figure supplement 8c), and Emx1 and total protein stain as control (reviewers Figure 1b). [file elife-55199-data30.zip › WB original and labeled blots/Reviewer Figure 1b Total protein Stain labeled plot.tif]

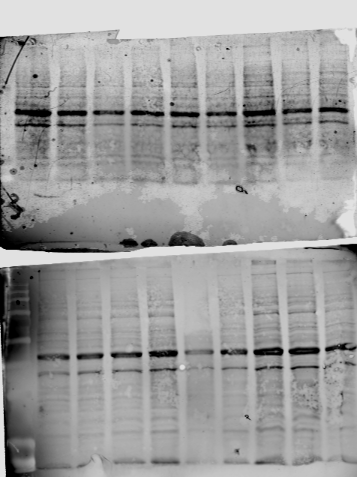

Supplement: Source data 30. — A zipped folder containing original and labeled bands photos for Western blots of Pum2 and tubulin as control (Figure 1—figure supplement 1e), human and mouse TDP-43 and total protein stain as control (Figure 1—figure supplement 8c), and Emx1 and total protein stain as control (reviewers Figure 1b). [file elife-55199-data30.zip › WB original and labeled blots/Reviewer Figure 1b total protein stain original plot.tif]
