# Supplementary material for: Pum2 and TDP-43 refine area-specific cytoarchitecture post-mitotically and modulate translation of Sox5, Bcl11b, and Rorb mRNAs in developing mouse neocortex
Source: eLife. 2022 Mar 9;11:e55199. doi: 10.7554/eLife.55199 (PMC8906809; doi:10.7554/eLife.55199)
Supplement: Transparent reporting form [file elife-55199-transrepform1.docx]

***eLife’s* transparent reporting form**

We encourage authors to provide detailed information *within their submission* to facilitate the interpretation and replication of experiments. Authors can upload supporting documentation to indicate the use of appropriate reporting guidelines for health-related research (see [EQUATOR Network](http://www.equator-network.org/%20)), life science research (see the [BioSharing Information Resource](https://biosharing.org/)), or the [ARRIVE guidelines](http://www.plosbiology.org/article/info:doi/10.1371/journal.pbio.1000412) for reporting work involving animal research. Where applicable, authors should refer to any relevant reporting standards documents in this form.

If you have any questions, please consult our Journal Policies and/or contact us: [editorial@elifesciences.org](mailto:editorial@elifesciences.org).

**Sample-size estimation**

- You should state whether an appropriate sample size was computed when the study was being designed
- You should state the statistical method of sample size computation and any required assumptions
- If no explicit power analysis was used, you should describe how you decided what sample (replicate) size (number) to use

Please outline where this information can be found within the submission (e.g., sections or figure legends), or explain why this information doesn’t apply to your submission:

The sample size was based on standard practice in the field.

**Replicates**

- You should report how often each experiment was performed
- You should include a definition of biological versus technical replication
- The data obtained should be provided and sufficient information should be provided to indicate the number of independent biological and/or technical replicates
- If you encountered any outliers, you should describe how these were handled
- Criteria for exclusion/inclusion of data should be clearly stated
- High-throughput sequence data should be uploaded before submission, with a private link for reviewers provided (these are available from both GEO and ArrayExpress)

Please outline where this information can be found within the submission (e.g., sections or figure legends), or explain why this information doesn’t apply to your submission:

For counting of immunostaining, retrograde labeling, FISH, Western blot, qPCR at least 3 animals (3-6 animals) of each genotype (biological replicates) were used. Each animal is from independant littermate and all controls for each n are control littermates.

For polysome profiling, at E13.5, E14.5, E18.5 and P0, cortices from respectively 4, 3, 2 or 1 animals were used as one biological replicate. 3 biological replicates were used in each experiment for each genotype.

For primary neurons experiments, cortices from almost 18 embryos from 3 different WT pregnant females were harvested and used as one biological replicate, this experiment was repeated for 3 times.

For CLIP, cortices from almost 10 embryos from 1-2 pregnant wt females were used and lysed in 5 ml lysis buffer, 500 ul of this lysis was used as one biological replicate for one immunoprecipitation. 3-6 biological replicates were used in 3 different technical replicates.

For in utero electroporations, 3 pregnant females were electroporated for each plasmid (combined to T-dimer red) and and its control GFP. Each animal come from an independent in utero electroporation experiment.

No data were excluded.

All this information is stated in Figure legends and methods.

**Statistical reporting**

- Statistical analysis methods should be described and justified
- Raw data should be presented in figures whenever informative to do so (typically when N per group is less than 10)
- For each experiment, you should identify the statistical tests used, exact values of N, definitions of center, methods of multiple test correction, and dispersion and precision measures (e.g., mean, median, SD, SEM, confidence intervals; and, for the major substantive results, a measure of effect size (e.g., Pearson's r, Cohen's d)
- Report exact p-values wherever possible alongside the summary statistics and 95% confidence intervals. These should be reported for all key questions and not only when the p-value is less than 0.05.

Please outline where this information can be found within the submission (e.g., sections or figure legends), or explain why this information doesn’t apply to your submission:

A two-tailed T-test was used for all immunostaining, retrograde labeling, FISH, and qPCR experiments following standard practice for this type of data.

A one-tailed T-test was used for qPCR on polysome profiling experiments, as we had a prior hypothesis based on protein staining and mRNA level data.

A one-tailed T-test was used for western blot for human and mouse TDP-43 as we had a prior hypothesis on over-expression in these mouse lines.

A Mann Whitney U Test was used for CLIP experiment as we deemed it to be most appropriate for this type of data.

Data are represented as means ± SEM for all experiments. P value: * p≤0.05, ** p≤0.01, ***p≤0.001

All this information is provided in figure legends and methods.

(For large datasets, or papers with a very large number of statistical tests, you may upload a single table file with tests, Ns, etc., with reference to sections in the manuscript.)

**Group allocation**

- Indicate how samples were allocated into experimental groups (in the case of clinical studies, please specify allocation to treatment method); if randomization was used, please also state if restricted randomization was applied
- Indicate if masking was used during group allocation, data collection and/or data analysis

Please outline where this information can be found within the submission (e.g., sections or figure legends), or explain why this information doesn’t apply to your submission:

This information doesn’t apply to our submission.

**Additional data files (“source data”)**

- We encourage you to upload relevant additional data files, such as numerical data that are represented as a graph in a figure, or as a summary table
- Where provided, these should be in the most useful format, and they can be uploaded as “Source data” files linked to a main figure or table
- Include model definition files including the full list of parameters used
- Include code used for data analysis (e.g., R, MatLab)
- Avoid stating that data files are “available upon request”

Please indicate the figures or tables for which source data files have been provided:

Source data are submitted with the manuscript.
